# Never use genes: terminology effects on public acceptance and implications for agricultural biotechnology

**DOI:** 10.1080/21645698.2026.2760092

**Published:** 2026-11-12

**Authors:** Hepeng Jia, Ruifen Zhang, Jingqun Fan, Zheng Yang, Jiaqi Zhang

**Affiliations:** a School of Communication, Soochow University, Suzhou, China; b School of Public Health, Soochow University, Suzhou, China; c Center for Bioscience Communication, Huazhong Agricultural University, Wuhan, China; d Dingduan News, Henan Daily Ltd., Zhengzhou, China

**Keywords:** Agricultural biotechnology, genetics knowledge, GM foods, naming effects, naturalness orientation, transgenic

## Abstract

Public acceptance remains a major social bottleneck in the diffusion of agricultural biotechnologies. Following the U.S. policy of mandating the labeling of genetically modified foods as “bioengineered” rather than using gene-explicit terminology, this study examines whether alternative Chinese names for agricultural biotechnologies shape public acceptance and related evaluations. In a one-way between-subjects online experiment, 1,217 Chinese participants were randomly assigned to one of four naming conditions: *biotechnology food*, *transgenic food*, *genetically modified food*, or *genetic engineering food*. While *biotechnology food* elicited higher acceptance, lower perceived risk, higher perceived benefits, and higher perceived naturalness, the three gene-explicit terms did not show significant differences in these dimensions. Moderated mediation analyses showed that the association between naming and acceptance was primarily through perceived benefits and, more conditionally, through perceived risk. Stronger naturalness orientation amplified naming differences in perceived risks and benefits and strengthened the association between perceived benefits and acceptance, whereas genetics knowledge played a more limited moderating role. The findings highlight the role of terminology in public evaluations of GM foods and suggest that responses to naming are sensitive to concerns about naturalness. For food labeling governance in China, terminology choices should be evaluated for their effects on consumer understanding, informed choice and acceptance.

## Introduction

Agricultural biotechnology is widely regarded as a promising tool for improving crop productivity, nutritional quality, and agricultural sustainability. Yet public acceptance remains a persistent bottleneck to the diffusion of the technology.^1–4^ This pattern is also evident in China, where both the general public and peasants remain cautious about genetically modified (GM) foods and crops. ^5–7^

Food labeling provides a means of communicating information about agricultural biotechnology to consumers.^8,9^ However, the issue is not only whether information is disclosed but also how the technology is named. In the United States, the National Bioengineered Food Disclosure Standard issued by the U.S. Department of Agriculture (USDA) established a mandatory national disclosure regime for GM products and standardized the regulatory term “bioengineered.”^10^ This development suggests that terminology has already become part of agricultural governance design rather than being merely a stylistic choice. Studies have likewise shown that the terms *genetic modification* and *genetic engineering* elicit more negative and less science-related associations than *biotechnology*. At the same time, GM food, genetically modified organisms (GMOs), and transgenic crops have been widely stigmatized.^11–13^

The issue is equally salient in China. The current Administrative Measures on Labeling Agricultural GMOs, enacted in 2002, require labeling for products included in the official catalog, and the rules explicitly prescribe label wording such as “transgenic (zhuanjiyin (转基因)) ××” or “raw material from transgenic ××.”^14^ A draft revision notified in 2023 proposed notable adjustments, including a 3% labeling threshold for listed products and restrictions on “non-GMO” claims.^15^ Although the labeling policy revision has not yet been finalized, recent national planning documents continue to call for faster industrial adoption of bio-breeding technologies.^16^ Together, these developments make naming a practical governance issue in China’s evolving agricultural biotechnology landscape.

Existing research has already shown that food labeling can affect consumer judgment, but most studies focus on label presence, information content, disclosure format, or consumer characteristics.^8,9,17,18^ Studies of cultivated meat have shown that alternative names can shape consumer evaluations of novel foods.^19,20^ These findings highlight the relevance of examining how alternative names influence responses to GM foods. More recent research likewise suggests that engineering-, science-, and design-related signifiers in disclosure statements can shape consumer perceptions of GM foods.^21^ Terminology may therefore function as an information cue: names that explicitly foreground gene-level intervention may heighten perceived human interference, unnaturalness, and risk, whereas broader terms such as biotechnology may attenuate those concerns.^6,11,21^ The International Service for the Acquisition of Agri-biotech Applications (ISAAA) has repeatedly used “biotech crops” and “biotech/GM crops” to refer to genetically modified crops. Biotechnology-related terminology also appears in media coverage and institutional discourse beyond ISAAA. Miller et al.,^22^ for example, identified “biotechnology/biotech,” alongside “genetically modified” and “genetically engineered,” among the most common terms used to introduce the technology in their sample of U.S. print-media coverage. The Food and Agriculture Organization of the United Nations^23^ also uses the broader category “agricultural biotechnology,” which includes GMOs. However, the circulation of these terms should not be taken to imply technical equivalence. Nelson^24^ highlighted the widespread but often imprecise use of “biotechnology” and “genetically modified” in academic and public discourse, warning that such terminology could reinforce misconceptions about transgenic agriculture.

Against this background, the present study examines whether alternative Chinese names for GM foods generate systematically different public responses. While the acronym GMO and the Chinese term zhuanjiyin (转基因, “transgenic”) are widely recognized as stigmatized,^12,25^ far less is known about whether the word “gene” and its various forms may elicit similarly negative public responses. Therefore, the current study compares a broader, less gene-explicit term, “*biotechnology food*,” with three more gene-explicit alternatives: “*transgenic food*,” “*genetic engineering food*,” and “*genetically modified food*” (literally translated as *jiyin gaizao* (基因改造)). The study investigates how these terms shape overall acceptance, perceived risks, perceived benefits, and perceived naturalness of the technology and the foods it produces. It further examines the risk–benefit mechanism through which alternative names may influence acceptance. By shifting attention to the naming of the technology itself, this study extends current research on food labeling and consumer acceptance to ongoing debates over how agricultural biotechnology should be communicated in China. The findings may also have implications for genome-edited foods, which could become increasingly popular in the near future.

## Literature Review

### Public Opposition, Stigma, and the Symbolic Burden of GM Terminology

Public attitudes toward GM foods and crops have been studied extensively for more than three decades.^3^ Research suggests that these attitudes are shaped not only by factual beliefs, but also by affective reactions, moral intuitions, perceived risks and benefits, trust, and broader judgments about food, technology, and nature.^3,26–29^ Sociodemographic characteristics form part of the context in which these judgments develop, and educational attainment may be associated with opportunities to encounter and interpret scientific information. Age may reflect cohort differences in prior exposure to GM controversies, accumulated experience with food technologies, and patterns of media use. Income may influence the material significance attached to expected benefits, costs, and affordability, although purchasing power alone cannot explain general attitudes toward a technology. However, none of these demographic characteristics have been found to decisively shape the public attitude. The relationships between these demographic factors and GM acceptance may vary across social and informational contexts. In the present study, these sociodemographic variables are therefore treated as background characteristics rather than as the focal mechanisms of the naming effect. More proximal bases of evaluation can include knowledge, values, institutional trust, affective responses, and risk–benefit appraisals through which people interpret a technology. Chinese research similarly shows that prior attitudes, affect, and perceived risks and benefits contribute to evaluations of GM food.^6,30^

One implication of this extensive literature is that terms such as “*GM food*” or “*GMO*” may no longer function as neutral technical descriptors. In public controversies, these terms have often been embedded in narratives emphasizing contamination, danger, and unnatural intervention.^12,31^ Sandman’s^32^ hazard–outrage framework highlights how public judgments of risk involve concerns about trust and control alongside assessments of potential harm. Research on agricultural biotechnology similarly shows that trust and outrage are associated with public attitudes largely through risk perceptions.^33^ From this perspective, names associated with controversy may evoke concerns that extend beyond their technical meanings.

This provides one possible explanation for the more favorable responses elicited by broader biotechnology terminology in previous research. Hallman et al.^11^ found more positive associations with biotechnology than with genetic modification or genetic engineering. Zahry and Besley^34^ likewise found that agricultural biotechnology attracted the greatest relative support, while both this term and genetic engineering elicited more favorable responses than genetic modification on several outcomes. These findings suggest that a broader naming contrast can coexist with differences among gene-related terms.

Explicit references to genes may make genetic intervention salient, while the complete term and information about the intervention can also shape evaluation. Concern may differ according to whether genetic material is transferred across species boundaries.^35^ Greater acceptance of genome-edited than genetically modified tomatoes has been observed, with perceived precision discussed as one possible explanation.^36^

Evidence from other comparisons is mixed. Participants receiving infographic or video information were willing to pay more for CRISPR orange juice than for GM orange juice.^17^ No statistically significant overall difference in willingness-to-pay discounts between CRISPR and GM foods was found, despite differences in willingness to consume them in several countries.^37^ These studies compare related technologies and information conditions, illustrating variation in evaluation without isolating the effect of wording alone.

For the present study, previous naming research provides a basis for expecting *biotechnology food* to elicit more favorable evaluations than the three gene-explicit alternatives. However, the available evidence does not establish a consistent ordering among *transgenic food*, *genetically modified food*, and *genetic engineering food* in the Chinese context. We therefore distinguish a directional hypothesis about the broader naming contrast from a research question about differences among the three gene-explicit terms:

H1. Compared with three gene-explicit terms—*transgenic food*, *genetically modified food*, and *genetic engineering food*—the broader term *biotechnology food* will elicit more favorable public responses, including higher acceptance, lower perceived risk, and more positive benefit-related evaluations.

RQ1. Do the gene-explicit terms—*transgenic food*, *genetically modified food*, and *genetic engineering food*—produce significantly different responses from one another in the Chinese context?

### Naturalness as a Key Evaluative Dimension

Among the evaluative dimensions that may be especially sensitive to naming, perceived naturalness deserves particular attention. Research on attitudes toward GM foods and crops has repeatedly shown that resistance to the technology is often tied to concerns about unnaturalness rather than to narrowly technical risk calculations alone.^3,38,39^ In China, the long-standing philosophical ideal of *tianren heyi*, or the harmony between humanity and nature, has also made Chinese people particularly attentive to naturalness in agriculture.^40^

If naming makes gene-level intervention more or less salient, then terminology may also shape whether a food or technology is perceived as natural or unnatural. A broader term, such as *biotechnology food*, may sound less intrusive and less explicitly interventionist than labels that directly foreground genes and engineering. By contrast, terms such as *genetic engineering* or *genetically modified* may more readily suggest deep human interference with natural processes. Under this reasoning, naming effects should not only influence evaluations of risk and benefit but also affect perceived naturalness itself.

This issue matters because naturalness is not merely an additional attitude dimension. In the GM food literature, it often serves as a moralized evaluative shortcut.^2,3^ A food or technology that seems less natural may be judged more suspicious, riskier, and less acceptable even in the absence of concrete evidence about its health or environmental effects.^41^ Accordingly, terms like *gene* that more explicitly foreground human manipulation may systematically reduce perceived naturalness.^42,43^

H2. The food will be perceived as more natural when described as than when described using one of the three gene-explicit terms.

RQ2. Does the perceived naturalness of the food differ across the three gene-explicit naming conditions?

### The Mediating Roles of Risk and Benefit Perceptions

A large body of research suggests that risk and benefit perceptions are among the strongest predictors of public acceptance of food technologies.^7,38,42^ Consumers do not usually support or reject agricultural biotechnology in the abstract; rather, they evaluate whether the technology appears beneficial, harmful, or both.^27,44^ In the Chinese context, Jin et al.^6^ found that acceptability of GM foods was strongly associated with perceived benefits, while general attitudes and affect also shaped benefit and risk judgments. More recently, Yang et al.^45^ showed that labeling effects on purchase intentions could operate through risk perception. These findings indicate that wording effects may influence support indirectly by altering how risky or beneficial the technology appears.

This logic is especially relevant for terminology. A broader and less gene-explicit term, such as *biotechnology food*, may increase support not because the public views it as a fundamentally different technology, but because it lowers perceived risk and/or elevates perceived benefits. Conversely, more gene-explicit terms may heighten perceptions of interference and uncertainty, thereby increasing perceived risk and reduce support.^46,47^ Under this reasoning, the effect of naming on technology acceptance should be mediated by evaluative judgments rather than operating as a purely direct effect.

The distinction between direct and indirect effects is theoretically important. If terminology operates through perceptions of risk and benefit, then naming is not merely a superficial linguistic difference. Instead, it affects acceptance by altering the interpretive lens through which the technology is understood. This would suggest that naming is part of the cognitive architecture of food acceptance rather than a peripheral communication choice.

H3a. The effect of naming on overall acceptance will be mediated by perceived risk, such that *biotechnology food* (vs. the gene-explicit terms) will indirectly increase acceptance by reducing perceived risk.

H3b. The effect of naming on overall acceptance will be mediated by perceived benefits, such that *biotechnology food* (vs. the gene-explicit terms) will indirectly increase acceptance by increasing perceived benefits.

Because risk and benefit perceptions are themselves interpretive judgments, the indirect effect of naming on overall acceptance may vary across individuals. The next section, therefore, considers whether some factors condition the risk–benefit pathways through which naming influences overall acceptance.

### Naturalness Orientation and Genetics Knowledge as Moderators of the Risk–Benefit Pathways

Although previous studies suggest that terminology may shape evaluations of food technologies, the magnitude of these effects may vary across individuals.^19,48–50^ It is therefore necessary to examine how people differ in translating naming cues into perceived risks and perceived benefits, and in the connection between these evaluative judgments and acceptance. Individual characteristics may either moderate the direct connection between naming and acceptance or adjust one or more segments of the indirect process through perceived risk/benefit. Naturalness orientation and knowledge of genetics are particularly plausible boundary conditions because both have been widely examined as predictors of attitudes toward GM foods and crops.^33,51^

Naturalness orientation is one especially plausible moderator. Individuals who place greater value on preserving nature from human interference tend to respond more negatively to food technologies perceived as artificial or overly interventionist.^41,42^ Accordingly, gene-explicit labels may be more likely to activate concerns about human interference among those with a stronger naturalness orientation, thereby altering the extent to which naming shapes perceived risk and perceived benefit. Naturalness orientation may also condition the extent to which these appraisals translate into overall acceptance. At the same time, Chinese evidence suggests that naturalness-related judgments do not always translate straightforwardly into acceptance of GM foods and crops, so the precise stage and direction of moderation remain open questions.^6,52^

Genetics knowledge may also condition this appraisal process, but its role is theoretically mixed.^53–55^ On the one hand, greater knowledge may help consumers understand the technologies described by different terms and assess the information provided, potentially reducing their reliance on naming cues.^8,56^ On the other hand, knowledge may not override affective, normative, or symbolic interpretations triggered by naming.^57–59^ Prior work further suggests that knowledge can shape risk–benefit appraisal in different ways across studies.^60,61^ Given these mixed expectations, the present study treats genetics knowledge as an exploratory moderator of the risk–benefit pathways linking naming to overall acceptance.

RQ3a. Does naturalness orientation moderate naming effects on public responses to these foods either directly or via the risk–benefit pathways?

RQ3b. Does genetics knowledge moderate naming effects on public responses to these foods either directly or via the risk–benefit pathways?

Figure 1 presents the conceptual model. It depicts naturalness orientation and genetics knowledge as exploratory moderators of the risk–benefit pathways and the direct path through which naming may influence overall acceptance.

**Figure 1.**
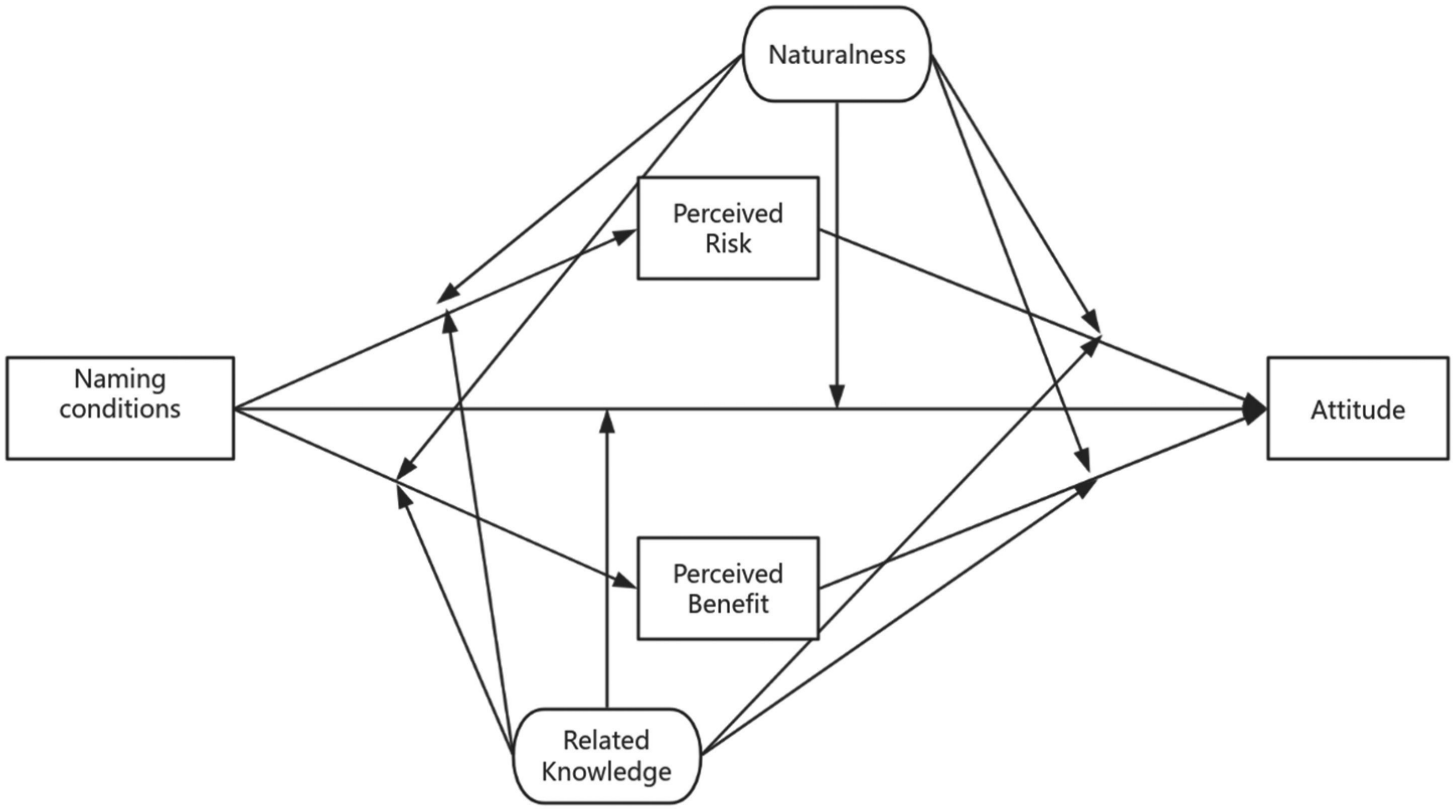
Conceptual model of moderated mediation linking naming to overall acceptance.

## Materials and Methods

### Experimental Design and Sample

This study employed a one-way between-subjects experiment in which food-technology naming condition served as the independent variable. While a survey experiment examining responses to a technological name does not establish whether the observed effects will extend to marketplace behavior, it can isolate naming effects under controlled conditions. The design can also provide insights into evaluative processes that may inform purchasing decisions, consistent with the approach taken in similar experimental studies.^34,62^

Participants were randomly assigned to one of four naming conditions: *biotechnology food*, *transgenic food*, *genetically modified food*, and *genetic engineering food*. Across conditions, all message content and question wording were identical except for the target term used to name the technology. The design, therefore, isolates the effect of terminology on overall acceptance, perceived risk, perceived benefits, and perceived naturalness. Participants evaluated only the term assigned to their condition and were not asked to choose among alternative names.

This study strictly adheres to research ethics standards and effectively protects the legitimate rights, privacy and physical and mental health of research subjects. The research protocol has obtained official approval from the administrative department of the researcher’s affiliated college, ensuring that the research content and process comply with the college’s research management regulations and academic norms.

We recruited 1,356 Chinese participants via the online survey platform WJX.cn in May 2024 for a web-based experiment (the platform charge was 19,622 yuan, including service fees). Random assignment was implemented automatically by the survey platform.

Before analysis, the data were screened for quality. Cases were excluded if they showed excessive missingness, unrealistically short completion times, or clear signs of inattentive or random responding. After data cleaning, 139 cases were removed, yielding 1,217 respondents for the ANOVA analyses. Group sizes were generally balanced, with 305 participants in the *biotechnology* condition, 296 in the *transgenic* condition, 316 in the *genetically modified* condition, and 300 in the *genetic engineering* condition. Table 1 presents the demographic characteristics of the total sample and each experimental group. Overall, the sample was relatively balanced in gender, and the demographic distributions across the four groups were broadly similar, suggesting that random assignment produced generally comparable groups. The conditional process analyses were based on 1,215 cases because two observations had missing data on variables included in the PROCESS models and were dropped by listwise deletion.

**Table 1.** Demographic characteristics of the total sample and by experimental condition.

Variable	Category	Total (*N* = 1217)	BF (*n* = 305)	TF (*n* = 296)	GMF (*n* = 316)	GEF (*n* = 300)
Gender	Male	616 (50.6)	151 (49.5)	143 (48.3)	169 (53.5)	153 (51.0)
	Female	600 (49.3)	154 (50.5)	153 (51.7)	147 (46.5)	146 (48.7)
	Other	1 (0.1)	—	—	—	1 (0.3)
Age	18–25	188 (15.5)	38 (12.5)	43 (14.5)	54 (17.1)	53 (17.8)
	26–36	733 (60.3)	197 (64.6)	173 (58.4)	190 (60.1)	173 (58.1)
	37–47	214 (17.6)	48 (15.7)	59 (19.9)	55 (17.4)	52 (17.4)
	48–58	64 (5.3)	17 (5.6)	20 (6.8)	12 (3.8)	15 (5.0)
	59–70	16 (1.3)	5 (1.6)	1 (0.3)	5 (1.6)	5 (1.7)
Education	Below high school	18 (1.5)	4 (1.3)	4 (1.4)	5 (1.6)	5 (1.7)
	High school	46 (3.8)	13 (4.3)	12 (4.1)	10 (3.2)	11 (3.7)
	Junior college	231 (19.0)	51 (16.7)	51 (17.2)	57 (18.0)	72 (24.0)
	Bachelor’s degree	806 (66.2)	205 (67.2)	197 (66.6)	215 (68.0)	189 (63.0)
	Master’s degree	107 (8.8)	29 (9.5)	31 (10.5)	26 (8.2)	21 (7.0)
	Doctoral degree	7 (0.6)	2 (0.7)	1 (0.3)	2 (0.6)	2 (0.7)
	Other	2 (0.2)	1 (0.3)	—	1 (0.3)	—
Monthly income	<1000	44 (3.6)	7 (2.3)	9 (3.0)	12 (3.8)	16 (5.3)
	1000–2000	43 (3.5)	8 (2.6)	12 (4.1)	13 (4.1)	10 (3.3)
	2000–4000	221 (18.2)	59 (19.3)	43 (14.5)	65 (20.6)	54 (18.0)
	4000–6000	344 (28.3)	101 (33.1)	79 (26.7)	78 (24.7)	86 (28.7)
	6000–8000	266 (21.9)	55 (18.0)	72 (24.3)	69 (21.8)	70 (23.3)
	8000– 10,000	181 (14.9)	55 (18.0)	46 (15.5)	43 (13.6)	37 (12.3)
	>10000	118 (9.7)	20 (6.6)	35 (11.8)	36 (11.4)	27 (9.0)

Note: Values in the table represent the number of participants, with percentages presented in parentheses. BF = *biotechnology food*; TF = *transgenic food*; GMF = *genetically modified food*; GEF = *genetic engineering food*.

As shown in Table 1, the sample was approximately balanced by gender (50.6% male and 49.3% female), with most respondents aged 26–36 years (60.3%) and holding a bachelor’s degree (66.2%). The largest monthly income category was 4,000–6,000 yuan (28.3%). Descriptively, the distributions of gender, age, education, and income were broadly similar across the four naming conditions. These demographic variables were included as covariates in the conditional process analyses.

### Measures

For all measures, the target term varied by experimental condition. The examples below use “biotechnology food”; in the other conditions, this phrase was replaced with “transgenic food,” “genetically modified food,” or “genetic engineering food.”

Overall acceptance was measured using a single item that asked whether participants supported or opposed the use of the target technology in agriculture and food production. Responses ranged from 1 (strongly oppose) to 7 (strongly support), with higher scores indicating greater acceptance.

Perceived benefits were measured with two items assessing the perceived health consequences and the broader balance of benefits and harms of the target food, including agricultural, economic, and environmental considerations. Responses ranged from 1 (strongly oppose) to 7 (strongly support). One item was reverse-coded so that higher values indicated greater perceived benefits on both items. The two items were averaged to form a perceived-benefits index (Cronbach’s α = .716).

Perceived risk was measured using a single item asking participants how harmful they thought the target food was. Participants responded using an online slider ranging from 1 (not harmful at all) to 10 (extremely harmful), with higher scores indicating greater perceived risk.

Perceived naturalness was also assessed using a single item asking participants how natural they considered the target food. Participants responded using an online slider ranging from 1 (not natural at all) to 10 (extremely natural), with higher scores indicating greater perceived naturalness.

Naturalness orientation was measured with four items adapted from Peters et al.^39^ The items assessed the extent to which participants valued naturalness in food and were concerned about human intervention in nature. Responses ranged from 1 (strongly disagree) to 5 (strongly agree), with higher scores indicating a stronger naturalness orientation. The items were averaged to create a naturalness-orientation index. Internal consistency was modest (Cronbach’s α = .601).

Genetics knowledge was measured using eight factual true/false items drawn from prior research.^63,64^ Correct answers were coded as 1 and incorrect answers as 0. The items were summed to form a knowledge index, with higher values indicating greater genetics knowledge. To minimize possible priming effects, the naturalness-orientation and genetics-knowledge measures were presented after the four focal outcome variables. Age, gender, income, and educational level were also recorded and were included as covariates in the conditional process models.

### Data Analysis

Data analysis proceeded in three stages. First, exploratory analyses examined sociodemographic differences in overall acceptance, perceived risk, perceived benefits, and perceived naturalness within each of the four naming conditions. Subgroup means and standard deviations were calculated by gender, age (18–25, 26–36, 37–47, and 48–70 years), and education (high school or below, junior college, bachelor’s degree, and postgraduate education). Respondents with less than a high school education and those who had completed high school were combined into the “high school or below” category, while respondents with master’s or doctoral degrees were combined into the “postgraduate education” category. These adjacent educational levels were combined because the categories below high school and at the doctoral level contained few respondents within each naming condition, limiting the precision of separate subgroup comparisons. Gender differences were tested using two-sided Welch’s independent-samples t-tests, while age and educational differences were assessed with Welch’s one-way ANOVAs. The resulting 48 overall tests, comprising four naming conditions, three demographic variables, and four outcomes, were treated as a single family of comparisons. *p*-values were adjusted using Holm’s sequential procedure to control the family-wise error rate at .05.^65^ Games–Howell post hoc tests characterized age and educational differences that remained significant after adjustment, with pairwise comparisons adjusted within each corresponding demographic comparison set. Unadjusted and Holm-adjusted *p*-values for all tests are reported in Table S1.

Second, one-way ANOVAs were used to examine whether the four naming conditions differed in overall acceptance, perceived risk, perceived benefits, and perceived naturalness. Bonferroni-adjusted pairwise comparisons were then used to identify specific differences between the biotechnology condition and each gene-explicit condition, as well as differences among the three gene-explicit terms. These analyses were used to test H1, H2, RQ1, and RQ2.

Third, a moderated mediation analysis was conducted using Hayes’s PROCESS macro model 76 with 5,000 bootstrap samples. To examine the broader naming contrast articulated in H1, the conditional process analysis focused on the theoretically central contrast between biotechnology food (coded as 1) and the three gene-explicit terms combined (coded as 0). Perceived risk and perceived benefits were entered as parallel mediators. Naturalness orientation and genetic knowledge were specified as moderators of the paths from naming to the mediators, from the mediators to overall acceptance, and of the direct path from naming to overall acceptance. Age, gender, income, and education were included as covariates. Continuous moderators and mediators involved in interaction terms were mean-centered before estimating the conditional process model. These analyses were used to test H3a, H3b, RQ3a, and RQ3b.

## Results

### Sociodemographic Differences Within Naming Conditions

Six of the 48 overall comparisons yielded unadjusted *p*-values below .05. After Holm correction, two differences remained significant, both concerning perceived naturalness in the *genetic engineering food* condition. Perceived naturalness differed across age groups, Welch’s *F* (3, 73.09) = 7.10, Holm-adjusted *p* = .014, and educational levels, Welch’s *F* (3, 48.31) = 6.81, Holm-adjusted *p* = .030. No other overall comparison remained significant after adjustment (Table S1).

Follow-up Games–Howell comparisons showed higher naturalness ratings among respondents aged 37–47 and 48–70 than among those aged 18–25 (*p* = .019 and *p* = .001, respectively). Respondents aged 48–70 also reported higher ratings than those aged 26–36 (*p* = .009). For education, respondents with junior college qualifications or bachelor’s degrees reported higher naturalness ratings than those with postgraduate education (*p* < .001 and *p* = .006, respectively).

### Naming Effects Across Conditions

#### Naming Effects on Acceptance, Perceived Risk, and Benefits

H1 predicted that, compared with the three gene-explicit terms, the broader term “*biotechnology food*” would elicit more favorable public responses, including higher acceptance, lower perceived risk, and more positive benefit-related evaluations. One-way ANOVAs showed significant effects of naming condition on acceptance (*F* (3, 1213) = 23.14, *p* < .001, η^2^ = .054); perceived risk (*F* (3, 1213) = 10.84, *p* < .001, η^2^ = .026); perceived benefits (*F* (3, 1213) = 41.32, *p* < .001, η^2^ = .093).

The means and standard deviations by naming condition are summarized in Table 2. For overall acceptance, participants in the *biotechnology food* condition reported higher acceptance (*M* = 4.71, *SD* = 1.35) than those in the *transgenic food* condition (*M* = 3.86, *SD* = 1.65), the *genetically modified food* condition (*M* = 3.81, *SD* = 1.57), and the *genetic engineering food* condition (*M* = 3.92, *SD* = 1.61). Bonferroni-adjusted pairwise comparisons showed that *biotechnology food* was rated significantly higher than *transgenic food* (*MD* = 0.847, *SE* = 0.126, *p* < .001, 95% CI [0.51, 1.18]), *genetically modified food* (*MD* = 0.898, *SE* = 0.124, *p* < .001, 95% CI [0.57, 1.23]), and *genetic engineering food* (*MD* = 0.788, *SE* = 0.126, *p* < .001, 95% CI [0.46, 1.12]).

**Table 2.** Means with standard deviations by naming condition.

Variable	BF	TF	GMF	GEF
Acceptance	4.71 (1.35)	3.86 (1.65)	3.81 (1.57)	3.92 (1.61)
Perceived risk	5.46 (2.21)	6.39 (2.37)	6.42 (2.40)	6.16 (2.51)
Perceived benefits	4.95 (1.47)	3.79 (1.69)	3.63 (1.65)	3.93 (1.71)
Perceived naturalness	4.89 (2.39)	3.54 (2.57)	3.51 (2.44)	3.55 (2.54)

Note: Values are means with standard deviations in parentheses. BF = *biotechnology food*; TF = *transgenic food;* GMF = *genetically modified food*; GEF = *genetic engineering food*.

For perceived risk, participants perceived *biotechnology food* as less risky (*M* = 5.46, *SD* = 2.21) than *transgenic food* (*M* = 6.39, *SD* = 2.37), *genetically modified food* (*M* = 6.42, *SD* = 2.40), and *genetic engineering food* (*M* = 6.16, *SD* = 2.51). Bonferroni-adjusted comparisons indicated that *biotechnology food* produced significantly lower risk perceptions than *transgenic food* (*MD* = −0.933, *SE* = 0.194, *p* < .001, 95% CI [−1.44, −0.42]), *genetically modified food* (*MD* = −0.965, *SE* = 0.191, *p* < .001, 95% CI [−1.47, −0.46]), and *genetic engineering food* (*MD* = −0.708, *SE* = 0.193, *p* = .002, 95% CI [−1.22, −0.20]).

For perceived benefits, participants in the *biotechnology food* condition reported higher perceived benefits (*M* = 4.95, *SD* = 1.47) than those in the *transgenic food* condition (*M* = 3.79, *SD* = 1.69), the *genetically modified food* condition (*M* = 3.63, *SD* = 1.65), and the *genetic engineering food* condition (*M* = 3.93, *SD* = 1.71). Bonferroni-adjusted pairwise comparisons showed that *biotechnology food* was rated as significantly more beneficial than *transgenic food* (*MD* = 1.167, *SE* = 0.133, *p* < .001, 95% CI [0.815, 1.519]), *genetically modified food* (*MD* = 1.327, *SE* = 0.131, *p* < .001, 95% CI [0.981, 1.674]), and *genetic engineering food* (*MD* = 1.026, *SE* = 0.133, *p* < .001, 95% CI [0.675, 1.377]).

Taken together, these results support H1. Compared with the three gene-explicit terms, *biotechnology food* elicited higher acceptance, lower perceived risk, and higher perceived benefits.

#### Differences Among the Three Gene-Explicit Terms in Acceptance, Perceived Risk and Benefits

RQ1 asked whether the three gene-explicit terms—*transgenic food*, *genetically modified food*, and *genetic engineering food*—would produce significantly different responses from one another. Bonferroni-adjusted pairwise comparisons showed that the three gene-explicit terms were generally evaluated similarly.

For overall acceptance, no significant differences were found among the three gene-explicit terms. *Transgenic food* did not differ from *genetically modified food* or *genetic engineering food*, and *genetically modified food* did not differ from *genetic engineering food* (all Bonferroni-adjusted *ps* = 1.000).

For perceived risk, the three gene-explicit terms also did not differ significantly from one another (all Bonferroni-adjusted *ps* = 1.000).

For perceived benefits, Bonferroni-adjusted comparisons again showed no significant differences among the three gene-explicit terms. *Transgenic food* did not differ from *genetically modified food* or *genetic engineering food* (both adjusted *ps* = 1.000), and *genetically modified food* did not differ from *genetic engineering food* (adjusted *p* = .132).

#### Naming Effects on Perceived Naturalness

H2 predicted that *biotechnology food* would be perceived as more natural than the three gene-explicit terms. A one-way ANOVA showed a significant effect of naming condition on perceived naturalness (*F* (3, 1213) = 22.73, *p* < .001, η^2^ = .053). Participants in the *biotechnology food* condition perceived the food as more natural (*M* = 4.89, *SD* = 2.39) than those in the *transgenic food* condition (*M* = 3.54, *SD* = 2.57), the *genetically modified food* condition (*M* = 3.51, *SD* = 2.44), and the *genetic engineering food* condition (*M* = 3.55, *SD* = 2.54).

Bonferroni-adjusted pairwise comparisons showed that *biotechnology food* was rated as significantly more natural than *transgenic food* (*MD* = 1.351, *SE* = 0.203, *p* < .001, 95% CI [0.82, 1.89]), *genetically modified food* (*MD* = 1.385, *SE* = 0.200, *p* < .001, 95% CI [0.86, 1.91]), and *genetic engineering food* (*MD* = 1.345, *SE* = 0.202, *p* < .001, 95% CI [0.81, 1.88]).

#### Differences Among Gene-Explicit Terms in Perceived Naturalness

RQ2 asked whether the three gene-explicit terms differed in perceived naturalness. Bonferroni-adjusted pairwise comparisons showed no significant differences among the three gene-explicit terms.

*Transgenic food* did not differ significantly from *genetically modified food* (*p* = 1.000) or *genetic engineering food* (*p* = 1.000). *Genetically modified food* did not differ from *genetic engineering food* (*p* = 1.000) either.

Overall, *biotechnology food* elicited higher overall acceptance, higher perceived benefits and perceived naturalness, and lower perceived risk than the three gene-explicit conditions. No statistically significant differences among the three gene-explicit conditions were detected on these outcomes.

### Moderated Mediation Analysis

We next estimated the conditional process model with 1,215 complete cases. Because the continuous moderators and mediators were mean-centered, lower-order coefficients represent effects at the mean levels of the centered variables.

#### Effects of Naming on Perceived Risk

The model predicting perceived risk was significant (*R*^2^ = .0425, *F* (9, 1205) = 5.9381, *p* < .001). Naming condition significantly predicted perceived risk at the mean levels of the moderators, indicating that *biotechnology food* elicited lower perceived risk than the three gene-explicit terms (*B* = −0.8737, *SE* = 0.1563, *t* = −5.5886, *p* < .001, 95% CI [−1.1804, −0.5670]). The interaction between naming condition and naturalness orientation was significant, indicating that the risk-reducing effect of *biotechnology food* became stronger as naturalness orientation increased (*B* = −0.4526, *SE* = 0.1657, *t* = −2.7309, *p* = .0064, 95% CI [−0.7778, −0.1275]). By contrast, the interaction between naming condition and genetics knowledge was not significant (*p* = .1298).

Conditional effect estimates further illustrated this pattern. At the median level of genetics knowledge (0.5292), the effect of naming on perceived risk was not significant when naturalness orientation was low (*p* = .1876), but became significant when naturalness orientation was moderate (*B* = −0.7774, *SE* = 0.1668, *t* = −4.6620, *p* < .001, 95% CI [−1.1046, −0.4502]) and high (*B* = −1.2301, *SE* = 0.2234, *t* = −5.5067, *p* < .001, 95% CI [−1.6683, −0.7918]). Thus, *biotechnology food* generally reduced perceived risk relative to the gene-explicit terms, and this difference became larger among participants with a stronger naturalness orientation.

#### Effects of Naming on Perceived Benefits

The model predicting perceived benefits was also significant (*R*^2^ = .1162, *F* (9, 1205) = 17.5954, *p* < .001). The naming condition significantly predicted perceived benefits at the mean levels of the moderators, indicating that *biotechnology food* elicited greater perceived benefits than the three gene-explicit terms (*B* = 1.1879, *SE* = 0.1071, *t* = 11.0929, *p* < .001, 95% CI [0.9778, 1.3980]). The interaction between the naming condition and naturalness orientation was significant, indicating that the benefit-enhancing effect of *biotechnology food* increased as naturalness orientation increased (*B* = 0.2823, *SE* = 0.1135, *t* = 2.4863, *p* = .0130, 95% CI [0.0595, 0.5050]). The interaction between naming condition and genetics knowledge was not significant (*p* = .7307).

Conditional effects indicated that naming significantly influenced perceived benefits across all tested levels of the moderators. At the median level of genetics knowledge (0.5292), the effect of naming on perceived benefits was significant when naturalness orientation was low (*B* = 0.8852, *SE* = 0.1687, *t* = 5.2469, *p* < .001, 95% CI [0.5542, 1.2162]), moderate (*B* = 1.1675, *SE* = 0.1142, *t* = 10.2210, *p* < .001, 95% CI [0.9434, 1.3916]), and high (*B* = 1.4498, *SE* = 0.1530, *t* = 9.4752, *p* < .001, 95% CI [1.1496, 1.7500]). These results indicate that *biotechnology food* increased perceived benefits relative to the gene-explicit terms, and this advantage increased with a stronger naturalness orientation.

#### Effects of the Mediators on Overall Acceptance

The outcome model predicting acceptance was significant (*R*^2^ = .5967, *F* (15, 1199) = 118.2705, *p* < .001). The lower-order coefficient for the naming condition was not significant at the mean levels of the moderators (*p* = .8434). In addition, neither the interaction between naming condition and naturalness orientation nor the interaction between naming condition and genetics knowledge was significant, indicating that the direct effect of naming on acceptance did not vary by either moderator (*p* > .4930).

For the risk pathway, perceived risk negatively predicted acceptance at the mean levels of the moderators (*B* = −0.0718, *SE* = 0.0145, *t* = −4.9641, *p* < .001, 95% CI [−0.1002, −0.0434]). The interaction between perceived risk and naturalness orientation was not significant (*p* = .3312). The interaction between perceived risk and genetics knowledge was significant (*B* = −0.0179, *SE* = 0.0093, *t* = −1.9196, *p* = .0451, 95% CI [−0.0362, −0.0004]).

Conditional effects nevertheless showed that perceived risk was negatively associated with acceptance under most tested combinations of the moderators. At the median level of genetics knowledge (0.5292), the effect of perceived risk on acceptance was negative and significant when naturalness orientation was low (*B* = −0.0676, *SE* = 0.0218, *t* = −3.0984, *p* = .0020, 95% CI [−0.1104, −0.0248]), moderate (*B* = −0.0809, *SE* = 0.0159, *t* = −5.0963, *p* < .001, 95% CI [−0.1121, −0.0498]), and high (*B* = −0.0942, *SE* = 0.0201, *t* = −4.6937, *p* < .001, 95% CI [−0.1336, −0.0548]).

For the benefit pathway, perceived benefits positively predicted acceptance at the mean levels of the moderators (*B* = 0.6328, *SE* = 0.0211, *t* = 30.0196, *p* < .001, 95% CI [0.5914, 0.6741]). The interaction between perceived benefits and naturalness orientation was significant, indicating that the positive effect of perceived benefits on acceptance became stronger as naturalness orientation increased (*B* = 0.0857, *SE* = 0.0202, *t* = 4.2503, *p* < .001, 95% CI [0.0461, 0.1252]). By contrast, the interaction between perceived benefits and genetics knowledge was not significant (*p* = .7001).

At the median level of genetics knowledge (0.5292), the effect of perceived benefits on acceptance was positive and significant when naturalness orientation was low (*B* = 0.5476, *SE* = 0.0309, *t* = 17.7031, *p* < .001, 95% CI [0.4869, 0.6082]), moderate (*B* = 0.6332, *SE* = 0.0233, *t* = 27.2134, *p* < .001, 95% CI [0.5876, 0.6789]), and high (*B* = 0.7189, *SE* = 0.0306, *t* = 23.4655, *p* < .001, 95% CI [0.6588, 0.7790]). Thus, perceived benefits consistently promoted acceptance, and this positive association became stronger among participants with higher naturalness orientation.

#### Conditional Direct Effects

The direct effect of naming on acceptance was not significant at any tested combination of the moderators. For example, the direct effect was not significant at low naturalness orientation and low genetics knowledge (*p* = .3455), at moderate naturalness orientation and median genetics knowledge (*p* = .9965), and at high naturalness orientation and high genetics knowledge (*p* = .4882). These results indicate that naming did not exert a significant direct effect on acceptance once perceived risk and perceived benefits were included in the model.

#### Indirect Effects

As shown in Table 3 , the conditional indirect effect of naming on acceptance, mediated by perceived risk, varied across combinations of naturalness orientation and genetics knowledge. Overall, the risk-based indirect effect was not significant at low naturalness orientation, regardless of genetics knowledge. For example, when naturalness orientation was low, and genetics knowledge was at the median, the indirect effect through perceived risk was not significant (*B* = 0.0220, *BootSE* = 0.0179, 95% BootCI [−0.0074, 0.0631]). By contrast, the indirect effect became significant when naturalness orientation was moderate or high. For instance, when naturalness orientation was moderate and genetics knowledge was at the median, the indirect effect was significant (*B* = 0.0629, *BootSE* = 0.0191, 95% BootCI [0.0303, 0.1044]); when naturalness orientation was high and genetics knowledge was high, the indirect effect was also significant (*B* = 0.1200, *BootSE* = 0.0428, 95% BootCI [0.0507, 0.2169]). These results indicate that the mediating role of perceived risk was conditional and depended primarily on naturalness orientation, with a weaker and less conclusive role for genetics knowledge.

**Table 3.** Conditional indirect effects of naming on overall acceptance.

Indirect pathway	Naturalness orientation	Genetics knowledge	Effect	BootSE	95% BootCI
Naming → Perceived risk → Acceptance	−1.0257	−1.4708	0.0205	0.0210	[−0.0129, 0.0697]
	−1.0257	0.5292	0.0220	0.0179	[−0.0074, 0.0631]
	−1.0257	1.5292	0.0141	0.0245	[−0.0307, 0.0677]
	−0.0257	−1.4708	0.0495	0.0267	[0.0031, 0.1091]
	−0.0257	0.5292	0.0629	0.0191	[0.0303, 0.1044]
	−0.0257	1.5292	0.0610	0.0258	[0.0179, 0.1198]
	0.9743	−1.4708	0.0906	0.0454	[0.0169, 0.1919]
	0.9743	0.5292	0.1159	0.0369	[0.0555, 0.1999]
	0.9743	1.5292	0.1200	0.0428	[0.0507, 0.2169]
Naming → Perceived benefits → Acceptance	−1.0257	−1.4708	0.5027	0.0980	[0.3146, 0.7073]
	−1.0257	0.5292	0.4847	0.0874	[0.3145, 0.6558]
	−1.0257	1.5292	0.4754	0.1050	[0.2721, 0.6839]
	−0.0257	−1.4708	0.7587	0.0968	[0.5707, 0.9543]
	−0.0257	0.5292	0.7393	0.0733	[0.5937, 0.8813]
	−0.0257	1.5292	0.7292	0.0968	[0.5423, 0.9186]
	0.9743	−1.4708	1.0631	0.1459	[0.7740, 1.3438]
	0.9743	0.5292	1.0422	0.1206	[0.8014, 1.2743]
	0.9743	1.5292	1.0314	0.1382	[0.7611, 1.3005]

Note: Continuous variables involved in interaction terms were mean-centered before analysis. Naturalness orientation values of −1.0257, −0.0257, and 0.9743 and genetics knowledge values of −1.4708, 0.5292, and 1.5292 correspond to the 16th, 50th, and 84th percentiles used by PROCESS after mean-centering. An indirect effect is considered significant when the 95% bootstrap confidence interval does not include zero.

In contrast, the conditional indirect effect of naming on acceptance through perceived benefits was significant across all tested combinations of naturalness orientation and genetics knowledge. Even at the lowest tested values of both moderators, the indirect effect through perceived benefits was significant (*B* = 0.5027, *BootSE* = 0.0980, 95% BootCI [0.3146, 0.7073]). This effect increased as naturalness orientation increased. For example, when naturalness orientation was moderate and genetics knowledge was at the median, the indirect effect was significant (*B* = 0.7393, *BootSE* = 0.0733, 95% BootCI [0.5937, 0.8813]); when naturalness orientation was high and genetics knowledge was high, it increased to 1.0314 (*BootSE* = 0.1382, 95% BootCI [0.7611, 1.3005]).

These findings show that the benefit-based indirect effect was more robust than the risk-based indirect effect. Whereas the risk pathway emerged only under some moderator conditions, the benefit pathway remained significant across all tested conditions and strengthened as naturalness orientation increased.

### Summary

Overall, the results provide clear support for the main naming effect predicted in this study. H1 and H2 were supported: compared with the three gene-explicit terms, *biotechnology food* elicited higher acceptance, lower perceived risk, higher perceived benefits, and higher perceived naturalness. RQ1 and RQ2 showed that the three gene-explicit terms did not differ significantly from one another in acceptance, perceived risk, perceived benefits, or perceived naturalness.

For the mechanism, H3a received conditional support: the indirect effect through perceived risk was significant only under some moderator conditions, especially when naturalness orientation was moderate or high. H3b was supported: the indirect effect through perceived benefits was significant across all tested moderator combinations and was consistently larger than the risk-based indirect effect. For RQ3a, naturalness orientation significantly moderated the effects of naming on both perceived risk and perceived benefits and also strengthened the positive effect of perceived benefits on acceptance. For RQ3b, genetics knowledge did not significantly moderate the effects of naming on the mediators or the direct effect of naming on acceptance. However, it moderated the risk–acceptance path. Thus, naturalness orientation played the broader and more consistent moderating role, whereas genetics knowledge showed only limited and suggestive involvement in the risk pathway.

## Discussion and Conclusions

In this Chinese survey experiment, *biotechnology food* elicited greater acceptance, higher perceived benefits and naturalness, and lower perceived risk than *transgenic food*, *genetically modified food*, and *genetic engineering food*. No statistically significant differences were detected among the latter three terms. With the accompanying information held constant, this contrast shows that the name used to describe an agricultural technology can influence its evaluation. One possible explanation concerns the salience of genetic intervention. Whereas *biotechnology* refers broadly to technological applications involving biological processes, the other names explicitly foreground genes. Genetic essentialism provides a theoretical basis for this interpretation: when genes are understood as defining fundamental properties, reference to their manipulation may make food seem more fundamentally altered.^42^ The lower naturalness ratings elicited by the gene-explicit terms are consistent with this interpretation.

Gene-related wording also acquires meaning within a wider information environment. Research on GMO controversies highlights the roles of advocacy, media coverage, and disputes over scientific authority and governance.^25,31^ These influences may shape the associations attached to particular names, alongside consumers’ interpretations of the intervention described. The mixed evidence on genome editing illustrates why references to genetic intervention need not produce identical evaluations across technologies and contexts. Our experiment compared complete names under otherwise identical survey conditions; it did not measure campaign exposure or establish how the associations attached to those names originated.

The mediation analysis was consistent with the proposed risk–benefit account of naming effects. The positive indirect effect through perceived benefits was significant across all tested combinations of naturalness orientation and genetics knowledge and was numerically larger than the indirect effect through perceived risk. The risk-based indirect effect was significant at moderate and high levels of naturalness orientation, but not at the low level. No significant direct effect of naming on acceptance was detected after the two appraisals were included in the model. These findings are consistent with research linking food-technology acceptance to risk and benefit perceptions^51^ and identifying perceived risk as a mediator of responses to GM labeling.^45^ In the present experiment, benefit appraisals provided the more consistent pathway linking terminology to acceptance.

Respondents with a stronger naturalness orientation showed larger naming differences in perceived risk and benefits, alongside a stronger association between perceived benefits and acceptance. This extends earlier research linking appreciation of nature to attitudes toward food biotechnology^39^ by showing that naturalness orientation also conditions responses to how a technology is named. Together with the higher naturalness ratings elicited by “biotechnology food,” these findings suggest that resistance to GM foods may reflect concerns about perceived interference with nature without necessarily implying a general rejection of technological innovation. This highlights the importance of clarifying the production practices behind a label.

Organic labeling provides a useful comparison because organic food is associated with naturalness,^41^ and organic labels can influence healthiness judgments even when nutritional information is available.^66^ These findings suggest that production terms can carry evaluative meanings beyond descriptions of farming practices.

Husaini and Sohail^67^ questioned the categorical separation of GM crops from organic agriculture, while their later work explored combining genetic technologies with cultivation practices intended to reduce agrochemical inputs.^68,69^ Their proposed label, organically grown GM produce, explicitly brings together breeding and cultivation information.^68^ These proposals draw attention to the distinction between how a crop is bred and how it is grown. In our experiment, higher naturalness ratings for *biotechnology food* under otherwise identical information conditions indicate that impressions of naturalness are sensitive to terminology. The GM–organic comparison therefore raises the possibility that names foreground different aspects of food production when consumers judge naturalness.

Genetics knowledge, by contrast, showed a narrower and more selective moderating role. It strengthened only the negative association between perceived risk and acceptance. In other words, more knowledgeable respondents relied more heavily on risk appraisal when forming an overall judgment. This pattern is consistent with previous research showing that knowledge has mixed and often limited effects on attitudes toward GM technologies.^52,53,57^ One plausible interpretation is that genetics knowledge does not inoculate respondents against symbolic naming cues, but it enables more knowledgeable individuals to interpret and evaluate safety-related concerns and therefore treat perceived risk as a more consequential basis for acceptance decisions. This interpretation is also consistent with prior work showing that risk information can exert a stronger influence on attitudes toward GM foods among more knowledgeable respondents.^55^

The demographic composition of the sample also provides context for interpreting these findings. Previous Chinese research has documented differences in GM-food attitudes across age, education, and income groups, with higher education and income not necessarily accompanied by greater acceptance.^5,61^ Our supplementary analyses identified age and educational differences in perceived naturalness within the “genetic engineering food” condition, whereas no other demographic comparison remained significant after Holm adjustment. These findings suggest that demographic background may be relevant to how naturalness is judged within this condition, but because these were within-condition comparisons, they do not establish demographic moderation of naming effects. The limited representation of older and less-educated respondents further warrants caution in generalizing these exploratory findings.

Taken together, our results contribute to theoretical work on naming and framing, particularly in the agriculture. They extend previous word-association studies and recent naming-framing research to Chinese GM-food terminology, showing that a broad, less gene-explicit label can substantially improve initial evaluations. In contrast, different gene-explicit expressions converge in public responses.^11,19,21,62^ The moderated mediation analyses further show that individual differences did not significantly moderate the direct effect of naming on acceptance. Instead, they shaped specific parts of the risk–benefit process through which naming influenced acceptance.

Terminology deserves attention in agricultural biotechnology communication, but more favorable evaluations should not be equated with better-informed consumer choices. Jaffe and Kuzma^70^ argued that replacing familiar GM terminology with “bioengineered” could create confusion and undermine trust if perceived as obscuring information. Empirical studies have also identified limited familiarity with the term and limited attention to its disclosure among university students.^71,72^ At the same time, choice-experiment evidence indicates that responses vary across disclosure formats and information-seeking conditions.^8^ These findings caution against evaluating disclosure solely by its effect on acceptance. Its policy relevance doesn’t mean a less gene-explicit label is necessarily preferable.

Disclosure should also distinguish the technology applied to a source crop from the composition of the resulting food ingredient. FDA guidance emphasizes describing foods as derived from genetically engineered plants and ensuring that explanatory claims refer accurately to the relevant ingredients.^73^ Future experiments could examine whether combining alternative names with explicit source information and concrete benefit explanations improves comprehension, trust, and acceptance.

Besides, genome editing provides a useful comparison for interpreting naming effects. Hu et al.^17^ found that consumers receiving infographic or video information were willing to pay more for CRISPR orange juice than for GM orange juice. This finding indicates that evaluations can differ across technologies and information conditions. The pattern observed in this study of GM foods—less favorable responses to gene-explicit terms than to “biotechnology food”—may not extend to genome-edited foods.

The results also caution against assuming that greater knowledge will automatically increase support for GM foods or neutralize framing effects. For more knowledgeable audiences, the naming effect remains, but the resulting risk perception may become more consequential. Effective communication and public education should therefore combine accurate scientific explanation with clear, concrete, and credible discussion of safety evidence. At the same time, because the benefit pathway was the dominant route—and became even stronger among respondents with higher naturalness orientation—public communication may be more effective when it combines less gene-explicit terminology with a persuasive explanation of tangible public and societal benefits.

Several limitations should be acknowledged. First, the experiment tested single-term exposure under survey conditions. This setting differs from a real-world purchasing context. Real labels appear together with brands, package design, price, nutrition claims, and competing non-GM cues. Second, the experiment compared a limited set of complete names, including only one less gene-explicit term. It therefore does not isolate the contribution of the word “gene” from differences in familiarity, semantic scope, or prior associations, nor establish which terminology best supports informed choice in practice.

Third, the sample and regulatory context are Chinese, and the meanings of terms may vary across languages and policy systems. Fourth, the online sample contained a high proportion of younger and university-educated respondents. This composition limits the generalizability of the estimates to older and less-educated populations. Replication with samples that more closely reflect the demographic distribution of the Chinese adult population would help establish how broadly the observed effects apply.

Finally, future work should test the persistence of naming effects under repeated exposure. It should continue to examine risk and benefit perceptions as mediating mechanisms, alongside other factors that may condition these effects over time.

## Supplementary Material

supplementary material.docx

## Data Availability

The data presented in this study are available on request from the corresponding author due to ethic.
