# Supplementary material for: Never use genes: terminology effects on public acceptance and implications for agricultural biotechnology
Source: GM Crops Food. 2026 Nov 12;17(1):2760092. doi: 10.1080/21645698.2026.2760092 (PMC13761378; doi:10.1080/21645698.2026.2760092)
Supplement: Supplementary file 1 [file KGMC_A_2760092_SM9299.docx]

**Table S1. Sociodemographic comparisons within each naming condition**

| **Naming condition** | **Demographic variable** | **Acceptance** | **Perceived risk** | **Perceived benefits** | **Perceived naturalness** |
| --- | --- | --- | --- | --- | --- |
| BF (N = 305) | Gender | .971 (1.000) | .356 (1.000) | .595 (1.000) | .400 (1.000) |
| BF | Age | .800 (1.000) | .259 (1.000) | .488 (1.000) | .363 (1.000) |
| BF | Education | .684 (1.000) | .555 (1.000) | .808 (1.000) | .009 (.384) |
| TF (N = 296) | Gender | .425 (1.000) | .134 (1.000) | .908 (1.000) | .287 (1.000) |
| TF | Age | .002 (.109) | .050 (1.000) | .009 (.384) | .455 (1.000) |
| TF | Education | .529 (1.000) | .276 (1.000) | .732 (1.000) | .341 (1.000) |
| GMF (N = 316) | Gender | .121 (1.000) | .121 (1.000) | .164 (1.000) | .688 (1.000) |
| GMF | Age | .235 (1.000) | .189 (1.000) | .115 (1.000) | .158 (1.000) |
| GMF | Education | .597 (1.000) | .342 (1.000) | .794 (1.000) | .133 (1.000) |
| GEF (N = 300) | Genderᵃ | .095 (1.000) | .697 (1.000) | .428 (1.000) | .518 (1.000) |
| GEF | Age | .149 (1.000) | .677 (1.000) | .035 (1.000) | <.001 (**.014**) |
| GEF | Education | .582 (1.000) | .959 (1.000) | .555 (1.000) | .001 (**.030**) |

*Note.* Entries are unadjusted p-values, with Holm-adjusted p-values in parentheses. Bold values indicate Holm-adjusted p < .05. BF = biotechnology food; TF = transgenic food; GMF = genetically modified food; GEF = genetic engineering food.

ᵃ the gender comparison in the GEF condition included 299 respondents.
